# Inflammatory properties of microparticles in stored red blood cell transfusion products

**DOI:** 10.1186/cc13303

**Published:** 2014-03-17

**Authors:** M Straat, R Nieuwland, R Van Bruggen, N Juffermans

**Affiliations:** 1 Academic Medical Center, Amsterdam, the Netherlands; 2 Sanquin, Amsterdam, the Netherlands

## Introduction

Blood transfusion is associated with increased morbidity and mortality in the critically ill [1]. Adverse effects of transfusion may be mediated by changes in the blood product that accumulate with storage time [2]. The mechanisms, however, are largely unknown. Erythrocyte-derived microparticles (MPs) have been found in transfusion bags [3],[4] and their concentration increases with storage duration [5]. We hypothesize that accumulation of MPs during storage induces a proinflammatory state in the recipient.

## Methods

Microparticles were isolated by high-speed centrifugation from red blood cell transfusion bags stored for 41 or 42 days. Whole blood from healthy volunteers was incubated for 24 hours with supernatant from the bags either containing MPs or depleted from MPs. Controls were incubated with medium or LPS (10 ng/ml). TNFa, IL-10 and IL-6 were measured in supernatant by ELISA. Data are expressed as median and range.

## Results

MP-depleted supernatant induced a modest increase in median levels of TNFa (9.2 (2.3 to 22.2) pg/ml) and IL-6 (2,140.7 (1,507 to 2,199) pg/ml) compared with the negative controls (2.3 (2.3 to 2.3) pg/ ml and 94.4 (64.7 to 124.1) pg/ml, respectively), while IL-10 levels were not affected. Addition of MP-containing supernatant resulted in highly increased levels of TNFa (699 (687 to 742) pg/ml), IL-6 (37,443 (26,493 to 40,967) pg/ml) and IL-10 (1,201 (1,178 to 1,533) pg/ml) compared with negative controls. This MP-induced increase in cytokine production was comparable with the increase observed after the addition of LPS.

## Conclusion

Erythrocyte-derived MPs from aged red blood cell transfusion bags induce a strong inflammatory response *in vitro, *which is largely negated when MPs are removed. Whether MPs mediate adverse effects of blood transfusion in the critically ill remains to be determined.
